# Envonalkib versus crizotinib for treatment-naive ALK-positive non-small cell lung cancer: a randomized, multicenter, open-label, phase III trial

**DOI:** 10.1038/s41392-023-01538-w

**Published:** 2023-08-14

**Authors:** Yunpeng Yang, Jie Min, Nong Yang, Qitao Yu, Ying Cheng, Yanqiu Zhao, Manxiang Li, Hong Chen, Shou’an Ren, Jianying Zhou, Wu Zhuang, Xintian Qin, Lejie Cao, Yan Yu, Jian Zhang, Jianxing He, Jifeng Feng, Hao Yu, Li Zhang, Wenfeng Fang

**Affiliations:** 1 Department of Medical Oncology, Sun Yat-sen University Cancer Center, State Key Laboratory of Oncology in South China, Collaborative Innovation Center for Cancer Medicine, Guangzhou, 510060 China; 2 Department of Oncology, The Second Affiliated Hospital of Air Force Medical University, Xi’an, 710038 China; 3 Department of Medical Oncology, Lung Cancer and Gastrointestinal Unit, Hunan Cancer Hospital/The Affiliated Cancer Hospital of Xiangya School of Medicine, Central South University, Changsha, 410013 China; 4 Department of Medical Oncology of Respiratory, Affiliated Tumor Hospital of Guangxi Medical University, Guangxi, Nanning, 530021 China; 5 Department of Thoracic Medical Oncology, Jilin Provincial Cancer Hospital, Changchun, 130012 China; 6 Department of Medical Oncology, The Affiliated Cancer Hospital of Zhengzhou University, Zhengzhou, 450003 China; 7 Department of Respiratory, First Affiliated Hospital of Xian Jiaotong University, Xi’an, 710061 China; 8 Department of Respiratory and Critical Care Medicine, The First Affiliated Hospital of Chongqing Medical University, Chongqing, 400050 China; 9 Department of Respiratory, The First Hospital of Shanxi Medical University, Taiyuan, 030001 China; 10 Department of Respiratory Disease, The First Affiliated Hospital, Zhejiang University School of Medicine, Hangzhou, 310003 China; 11 Department of Medical Oncology, Fujian Cancer Hospital, Fuzhou, 350014 China; 12 The First Department of Oncology, The First Affiliated Hospital of Guangdong Pharmaceutical University, Guangzhou, 510699 China; 13 Department of Respiratory and Critical Care Medicine, Anhui Provincial Hospital, Hefei, 230000 China; 14 Department of Respiratory Medicine, Affiliated Cancer Hospital of Harbin Medical University, Harbin, 150000 China; 15 Department of Respiratory Medicine, The First Affiliated Hospital of Air Force Medical University, Xi’an, 710032 China; 16 Department of Cardiothoracic Surgery, The First Affiliated Hospital of Guangzhou Medical College, Guangzhou Research Institute of Respiratory Disease and China State Key Laboratory of Respiratory Disease, Guangzhou, 510120 China; 17 Department of Medical Oncology, The Affiliated Cancer Hospital of Nanjing Medical University, Jiangsu Cancer Hospital, Jiangsu Institute of Cancer Research, Nanjing, 210009 China; 18 Department of Biostatistics, Nanjing Medical University, Nanjing, 211166 China

**Keywords:** Lung cancer, Cancer

## Abstract

Anaplastic lymphoma kinase (ALK) rearrangements are present in about 5–6% of non-small cell lung cancer (NSCLC) cases and associated with increased risks of central nervous system (CNS) involvement. Envonalkib, a novel ALK inhibitor, demonstrated promising anti-tumor activity and safety in advanced ALK-positive NSCLC in the first-in-human phase I study. This phase III trial (ClinicalTrials.gov NCT04009317) investigated the efficacy and safety of first-line envonalkib in advanced ALK-positive NSCLC cases. Totally 264 participants were randomized 1:1 to receive envonalkib (*n* = 131) or crizotinib (*n* = 133). Median independent review committee (IRC)-assessed progression-free survival (PFS) times were 24.87 (95% confidence interval [CI]: 15.64–30.36) and 11.60 (95% CI: 8.28–13.73) months in the envonalkib and crizotinib groups, respectively (hazard ratio [HR] = 0.47, 95% CI: 0.34–0.64, *p* < 0.0001). IRC-assessed confirmed objective response rate (ORR) was higher (81.68% vs. 70.68%, *p* = 0.056) and duration of response was longer (median, 25.79 [95% CI, 16.53–29.47] vs. 11.14 [95% CI, 9.23–16.59] months, *p* = 0.0003) in the envonalkib group compared with the crizotinib group. In participants with baseline brain target lesions, IRC-assessed CNS-ORR was improved with envonalkib compared with crizotinib (78.95% vs. 23.81%). Overall survival (OS) data were immature, and median OS was not reached in either group (HR = 0.84, 95% CI: 0.48–1.47, *p* = 0.5741). The 12-month OS rates were 90.6% (95% CI, 84.0%–94.5%) and 89.4% (95% CI, 82.8%–93.6%) in the envonalkib and crizotinib groups, respectively. Grade ≥3 treatment-related adverse events were observed in 55.73% and 42.86% of participants in the envonalkib and crizotinib groups, respectively. Envonalkib significantly improved PFS and delayed brain metastasis progression in advanced ALK-positive NSCLC.

## Introduction

Lung cancer constitutes the most common malignant tumors around the world. Estimated 2,093,876 newly diagnosed lung cancer cases were reported in 2018 globally, with yearly age-standardized incidence rates of 31.5/100,000 in males and 14.6/100,000 in females.^1^ Lung cancer is also the leading cause of cancer death, with yearly age-standardized mortality rates of 27.1/100,000 in males and 11.2/100,000 in females.^1^ Non-small cell lung cancer (NSCLC) comprises 85–90% of all the lung cancer cases, usually affecting adults who smoke and those 65 years old or above.^2^ Despite the advances in management, the prognosis of NSCLC remains poor in the United States, with 5-year survival rates of 19%, 56%, 30% and 5% for all, localized diseases, regional diseases, and metastatic diseases, respectively.^3^

The oncogenic anaplastic lymphoma kinase (ALK) rearrangements occur in ~5–6% of NSCLC patients and are mostly observed in young individuals as well as in light or non-smokers with adenocarcinoma.^4,5^ Above 19 distinct ALK fusion partners have been identified so far.^6^ Cases with ALK rearrangements show poor prognosis,^6^ for insistence, a higher risk of central nervous system (CNS) involvement.^7^ Therefore, the enthusiasm for ALK as a target for cancer therapy is encouraging. CNS metastases can lead to substantial morbidity and decrease the quality of life.^8^ The management strategies for CNS metastases mainly rely on local therapies (surgery, whole-brain radiotherapy, and stereotactic radiosurgery), but the advent of small-molecule systemic therapies which can cross the blood-brain barrier has improved the prognosis of NSCLC patients with CNS involvement, including those with ALK-positive NSCLC.^9^

ALK inhibitors have become the standard therapeutic option for advanced ALK-positive NSCLC.^10^ Crizotinib, a first-generation ALK inhibitor, showed a response rate close to 75% and a median progression-free survival (PFS) of nearly 12 months. Unfortunately, eventually all the cases administered crizotinib would develop acquired resistance, generally within 1–2 years after the initiation of treatment.^11^ Second-generation (ceritinib, alectinib, brigatinib, and ensartinib) and third-generation (lorlatinib) ALK inhibitors were designed to overcome resistance to crizotinib and improve the management of CNS metastases, showing anti-tumor activity in crizotinib-refractory patients. Recently, randomized phase III trials have demonstrated that alectinib, brigatinib, ensartinib and lorlatinib can significantly improve the treatment response of CNS metastases and PFS in comparison with crizotinib as first-line treatment .^12–15^ The 12-month PFS rate of alectinib was 68.4%, vs. 48.7% for crizotinib.^12^ In addition, ALK variants do not affect the efficacy of alectinib in NSCLC.^16^ Ensartinib was shown to be particularly effective for the management of CNS progression, achieving an intracranial response rate of 64%, vs. 21% for crizotinib.^13^ Lorlatinib yielded a particularly low HR of 0.28 for disease progression or death compared with crizotinib.^14^ Still, treatment eventually fails, and the high incidence rates of treatment resistance and disease recurrence are the major issues currently,^9^ calling for novel molecules that could be effective options once resistance occurs or to prevent its development. Indeed, the clinical efficacy of all ALK inhibitors is the eventual development of multidrug resistance because of the selection pressure of the treatments on NSCLC cells.^17^ Such resistance can be attributed to the occurrence of secondary mutations in the ALK tyrosine kinase domain, amplification of ALK, drug efflux pumps, activated bypass signaling pathways, lineage changes, and primary ALK-tyrosine kinase inhibitor (TKI) resistance.^17^

Envonalkib (CT-711, TQ-B3139, CTTQ Pharmaceutical Group Co., Ltd., Nanjing, China) is a newly developed small-molecule TKI against ALK, c-Met, and ROS1, which has a potency of five times higher than that of crizotinib in enzymatic assays.^18^ It is potentially sensitive to ALK-resistance mutations, including L1152R, R1275Q, L1196M, and C1156Y.^18^ Preclinical data support the superiority of envonalkib over crizotinib in ALK-driven malignancies and c-Met activation causing acquired resistance.^18^ The pharmacokinetic profile of envonalkib showed dose proportionality from 200 to 600 mg twice a day (BID), but absorption saturation was observed at 800 mg, showing a plateauing area under the curve and peak concentration.^19^ The maximum tolerated dose of envonalkib was not reached in the first-in-human phase I study, and 600 mg BID was established as the recommended phase II dose for safety concerns. The phase I study reported that envonalkib at 200 mg BID and beyond was safe and effective in both treatment-naïve and previously treated ALK-positive NSCLC cases. In addition, envonalkib showed promising therapeutic effects for brain metastases.^19^ Indeed, in cases with brain target lesions, an intracranial objective response rate (ORR) was of 70% was observed, and the median intracranial PFS reached 15.9 months.^19^ No new safety signal was detected in the phase I study that could prevent further study of the drug.

The present randomized phase III study aimed to compare envonalkib and crizotinib for efficacy and safety in patients with treatment-naive advanced ALK-positive NSCLC and to examine the effect of envonalkib against CNS metastases.

## Results

### Screening

**Fig. 1:**
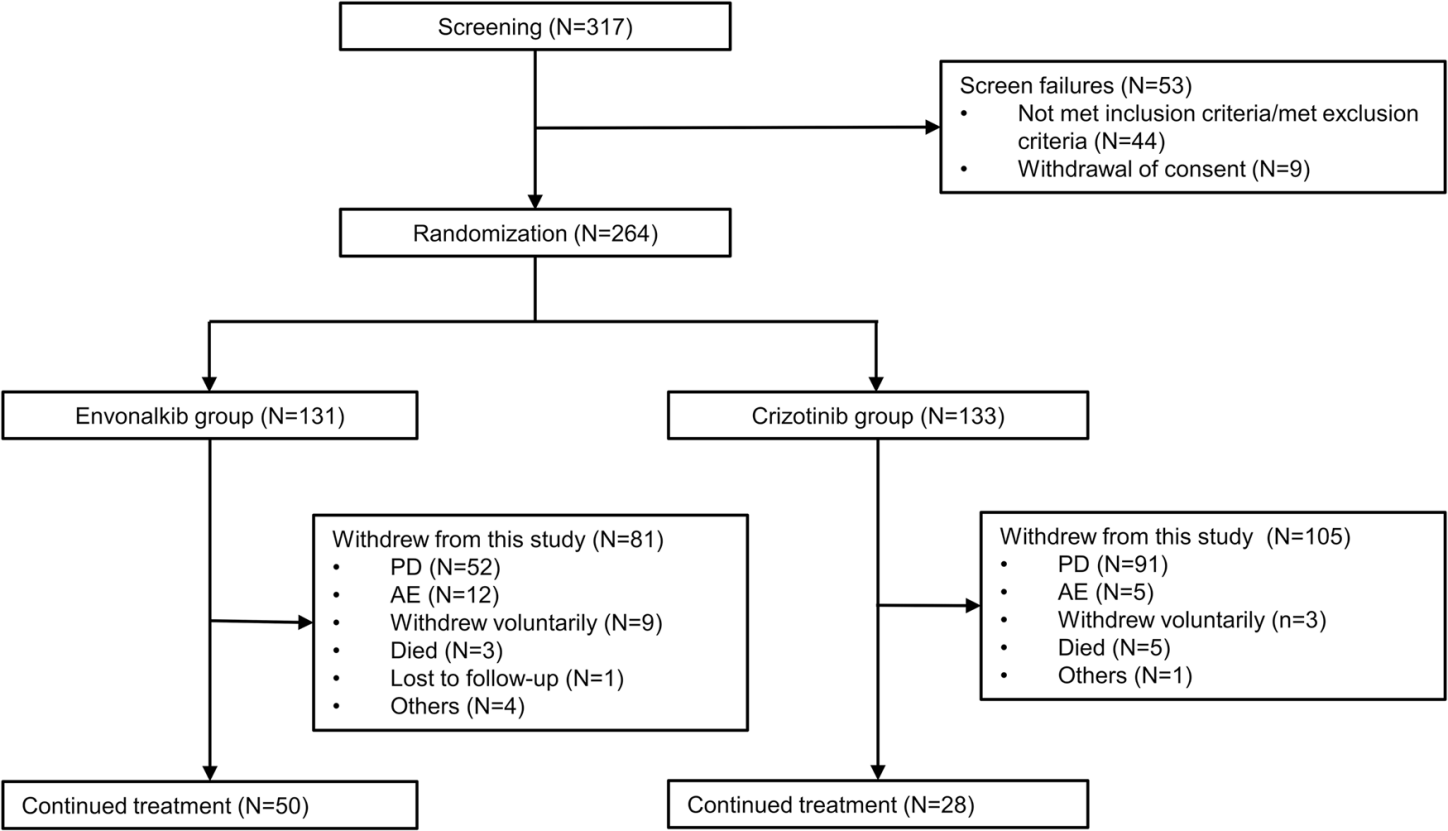
Study flowchart. PD progressive disease, AE adverse event, ITT intent-to-treat, SS safety set

Totally 317 NSCLC patients were screened, and 264 underwent randomization, including 131 and 133 in the envonalkib and crizotinib groups, respectively, both in the intent-to-treat (ITT) population and the safety set (SS). As of October 14, 2021, 135 PFS events were observed, and an interim analysis was performed. Considering the immature PFS data, follow-up for PFS was extended to August 31, 2022, when 81 and 105 participants had withdrawn from the study, while 50 and 28 were still on treatment in the envonalkib and crizotinib groups, respectively (Fig. 1).

### Baseline participant characteristics

The median age in the overall population was 53 years, with 85.23% of cases aged <65 years and 51.52% males. The majority of participants (93.94%) had stage IV disease, and 75.38% of participants had no previous chemotherapy. Participant characteristics were generally balanced between both groups, except that the proportions of participants with age ≥65 years (19.08% vs. 10.53%) and with an Eastern Cooperative Oncology Group performance status (ECOG PS) score of 1 (77.10% vs. 69.92%) were slightly higher in the envonalkib group (Table 1).

**Table 1:** Baseline characteristics of the study participants in the intent-to-treat (ITT) population

Characteristic	Envonalkib (*N* = 131)	Crizotinib (*N* = 133)
Age (years)		
Mean ± SD	53.4 ± 10.80	51.8 ± 10.83
Median (Q1, Q3)	53.0 (46.0, 62.0)	52.0 (46.0, 59.0)
<65	106 (80.92)	119 (89.47)
≥65	25 (19.08)	14 (10.53)
Sex (male)	68 (51.91)	68 (51.13)
ECOG		
0	30 (22.90)	40 (30.08)
1	101 (77.10)	93 (69.92)
Clinical stage		
IIIB	9 (6.87)	7 (5.26)
IV	122 (93.13)	126 (94.74)
Pathological type		
Adenocarcinoma	124 (94.66)	124 (93.23)
Others	7 (5.34)	9 (6.77)
Brain metastasis (IRC-assessed)		
Yes	43 (32.83)	45 (33.83)
No	88 (67.17)	88 (66.17)
Number of previous lines of chemotherapy		
0	98 (74.81)	101 (75.93)
1	33 (25.19)	32 (24.06)
Smoking history		
Never-smoker	90 (68.70)	84 (63.16)
Ex-smoker	41 (31.30)	46 (34.59)
Current smoker	0	3 (2.26)

*ECOG* Eastern Cooperative Oncology Group, *SD* standard deviation, *ITT* intent-to-treat

### Efficacy in the ITT population

**Fig. 2:**
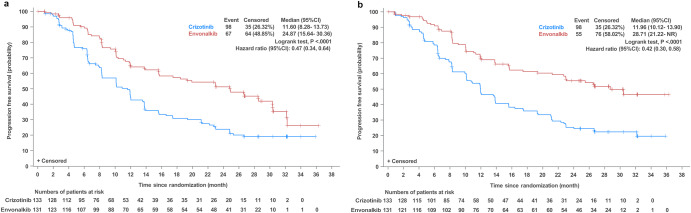
Progression-free survival assessed by the independent review committee (**a**) and the investigators (**b**) in the intent-to-treat population

**Fig. 3:**
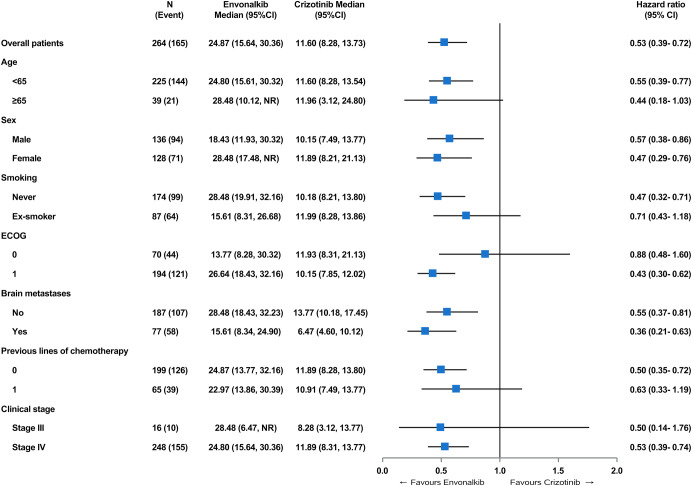
Subgroup analysis of independent review committee-assessed progression-free survival in the intent-to-treat population. ECOG Eastern Cooperative Oncology Group, HR hazard ratio, CI confidence interval

At the data cutoff date of August 31, 2022, the median follow-up durations in the envonalkib and crizotinib groups were 28.48 (95% CI, 26.58–30.32) and 28.55 (95% CI, 26.64–32.13) months, respectively. As shown in Fig. 2, the median independent review committee (IRC)-assessed PFS was significantly prolonged with envonalkib compared with crizotinib (median, 24.87 (95% confidence intervals [CI]: 15.64–30.36) vs. 11.60 (95% CI: 8.28–13.73) months; hazard ratio [HR] = 0.47, 95% CI: 0.34–0.64; log-rank *p* < 0.0001). The investigator-assessed PFS was also significantly longer in the envonalkib group than in the crizotinib group (median, 28.71 vs. 11.96 months; HR = 0.42, 95% CI: 0.30–0.58, log-rank *p* < 0.0001). The HR values favored envonalkib over crizotinib across all prespecified subgroups based on baseline patient features and stratification parameters (Fig. 3).

**Fig. 4:**
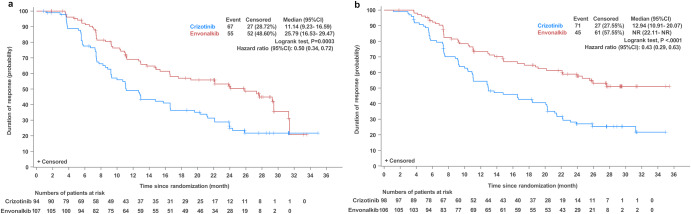
Independent review committee-assessed (**a**) and investigator-assessed (**b**) duration of response in the intent-to-treat population

IRC-assessed confirmed ORR was elevated in the envonalkib group in comparison with the crizotinib group (81.68% vs. 70.68%, *p* = 0.056) (Table 2). The median durations of response (DORs) were 25.79 (95% CI, 16.53–29.47) vs. 11.14 (95% CI, 9.23–16.59) months (HR = 0.50, 95% CI: 0.34–0.72, *p* = 0.0003) as assessed by the IRC, and not reached (NR) (95% CI, 22.11–NR) vs. 12.94 (95% CI, 10.91–20.07) months (HR = 0.43, 95% CI: 0.29–0.63, *p* < 0.0001) as assessed by the investigator (Fig. 4).

**Table 2:** IRC-assessed treatment response in the intent-to-treat (ITT) population

	Envonalkib (*N* = 131)	Crizotinib (*N* = 133)	*p* value
CR	0	0	
PR	107 (81.68)	94 (70.68)	
SD	13 (9.92)	24 (18.05)	
PD	6 (4.58)	11 (8.27)	
NE	5 (3.82)	4 (3.01)	
Confirmed ORR	107 (81.68)	94 (70.68)	0.056
95% CI	73.98–87.89	62.16–78.25	

*ORR* objective response rate, *CR* complete response, *PR* partial response, *SD* stable disease, *PD* progressive disease, *NE* not evaluable, *CI* confidence interval, *IRC* independent review committee

Data of the overall survival (OS) was immature, with deaths occurring in a total of 50 participants in the ITT population (23 [17.56%] and 27 [20.3%] participants in the envonalkib and crizotinib groups, respectively). The median OS was NR in both groups (HR = 0.84, 95% CI: 0.48–1.47; log-rank *p* = 0.5741). The 12-month OS rates were 90.6% (95% CI, 84.0–94.5%) and 89.4% (95% CI, 82.8–93.6%) in the envonalkib and crizotinib groups, respectively. The 24-month OS rates were 78.4% (95% CI, 67.7–85.9%) and 75.0% (95% CI, 64.3–82.9%) in the envonalkib and crizotinib groups, respectively.

### Efficacy in participants based on baseline intracranial lesions

Totally 88 (33.33%) participants had brain metastases at baseline, among whom 40 (15.15%) had intracranial target lesions. As shown in Supplementary Table 1 and Supplementary Fig. 1, in participants with baseline intracranial target lesions, the IRC-assessed CNS-ORR was significantly increased (78.95% vs. 23.81%), the IRC-assessed DOR was longer (median, 25.82 vs. 7.39 months; *p* = 0.0030), and the IRC-assessed CNS-time to progression (TTP) was longer (median, 26.68 vs. 6.34 months; *p* = 0.0008) in the envonalkib group compared with the crizotinib group. In participants with brain metastases at baseline, the benefit of envonalkib over crizotinib in IRC-assessed CNS-TTP (median, 30.32 vs. 8.28 months; *p* = 0.0001) was also observed (Supplementary Fig. 2). In addition, in participants without brain metastasis at baseline, IRC-assessed incidence rates of brain metastasis during the treatment were notably lower after treatment with envonalkib than crizotinib (2.15% vs. 11.70%, *p* = 0.0182). In participants with brain metastasis at baseline, the cumulative incidence of CNS progression was also lower with envonalkib than with crizotinib.

### Safety

Treatment durations were 531 (range, 2–1117) and 360 (range, 27–1100) days in the envonalkib and crizotinib groups, respectively. Treatment-emergent adverse events (TEAEs) occurred in 99.24% and 100% of participants in the envonalkib and crizotinib groups, respectively. The incidence rates of serious adverse events (SAEs) were 37.40% in the envonalkib group and 28.57% in the crizotinib group. Grade ≥3 treatment-related adverse events (TRAEs) occurred in 55.73% and 42.86% of participants in the envonalkib and crizotinib groups, respectively. The incidence rates of TRAEs leading to dose reduction and permanent discontinuation of the study drug were 33.59% and 6.11% in the envonalkib group, respectively, and 20.30% and 3.76% in the crizotinib group, respectively. The eight cases of envonalkib discontinuation due to TRAEs included two participants with gastrointestinal toxicity, one with arrhythmia, three with potential drug-induced liver injury, one with fatigue and peripheral neuropathy, and one with elevated creatine phosphokinase. The most common TEAEs (>10%) in both groups are shown in Table 3. The most common TEAEs in the envonalkib group were diarrhea (90.84%), vomiting (83.97%), elevated alanine transaminase (74.81%), nausea (70.23%), and elevated aspartate aminotransferase (69.47%).

**Table 3:** Safety profile and adverse events related to the treatment in the safety population

TEAE	Envonalkib (*N* = 131)		Crizotinib (*N* = 133)	
	Any grade	Grade ≥3	Any grade	Grade ≥3
All TEAEs	130 (99.24)	83 (63.36)	133 (100.00)	73 (54.89)
SAEs	49 (37.40)		38 (28.57)	
TRAEs	130 (99.24)	73 (55.73)	131 (98.50)	57 (42.86)
SAEs related to the study drug	33 (25.19)		11 (8.27)	
TRAEs leading to dose reduction	44 (33.59)	38 (29.01)	27 (20.30)	18 (13.53)
TRAEs leading to treatment suspension	3 (2.29)	2 (1.53)	2 (1.50)	1 (0.75)
TRAEs leading to treatment discontinuation	8 (6.11)	5 (3.82)	5 (3.76)	1 (0.75)
TRAEs leading to death	0	0	1 (0.75)	1 (0.75)
TRAEs of special interest	14 (10.69)	4 (3.05)	5 (3.76)	0
The most common TEAEs (>10% in both groups)				
Diarrhea	119 (90.84)	12 (9.16)	82 (61.65)	0
Vomiting	110 (83.97)	9 (6.87)	63 (47.37)	1 (0.75)
Elevated ALT	98 (74.81)	20 (15.27)	91 (68.42)	9 (6.77)
Nausea	92 (70.23)	4 (3.05)	58 (43.61)	0
Elevated AST	91 (69.47)	13 (9.92)	82 (61.65)	3 (2.26)
Loss of appetite	76 (58.02)	0	48 (36.09)	1 (0.75)
Sinus bradycardia	69 (52.67)	0	54 (40.60)	1 (0.75)
Hypoalbuminemia	63 (48.09)	2 (1.53)	50 (37.59)	1 (0.75)
ECG QT prolongation	42 (32.06)	19 (14.50)	21 (15.79)	8 (6.02)
Anemia	42 (32.06)	4 (3.05)	32 (24.06)	1 (0.75)
Elevated serum creatinine	41 (31.30)	0	27 (20.30)	0
Fatigue	41 (31.30)	2 (1.53)	32 (24.06)	1 (0.75)
Weight loss	41 (31.30)	2 (1.53)	13 (9.77)	0
Elevated serum CK-MB	38 (29.01)	7 (5.34)	34 (25.56)	4 (3.01)
Elevated serum CK	38 (29.01)	4 (3.05)	49 (36.84)	5 (3.76)
Elevated γ-GGT	36 (27.48)	12 (9.16)	27 (20.30)	2 (1.50)
Cough	33 (25.19)	0	28 (21.05)	0
Decreased PLT	34 (25.95)	4 (3.05)	4 (3.01)	0
Hypokalemia	32 (24.43)	9 (6.87)	6 (4.51)	0
Elevated serum LDH	31 (23.66)	0	45 (33.83)	0
Decreased WBC	31 (23.66)	4 (3.05)	57 (42.86)	10 (7.52)
Constipate	28 (21.37)	0	42 (31.58)	0
Proteinuria	25 (19.08)	0	18 (13.53)	0
Decreased neutrophil count	25 (19.08)	3 (2.29)	59 (44.36)	24 (18.05)
Hyperuricemia	24 (18.32)	0	15 (11.28)	0
Hypocalcemia	23 (17.56)	1 (0.76)	19 (14.29)	1 (0.75)
Weight gain	21 (16.03)	3 (2.29)	31 (23.31)	3 (2.26)
Dizziness	20 (15.27)	1 (0.76)	23 (17.29)	1 (0.75)
Upper respiratory infection	18 (13.74)	1 (0.76)	21 (15.79)	2 (1.50)
Peripheral edema	17 (12.98)	0	36 (27.07)	1 (0.75)
Insomnia	17 (12.98)	0	16 (12.03)	0
Elevated α-hydroxybutyrate dehydrogenase	14 (10.69)	0	18 (13.53)	0
Decreased lymphocyte count	14 (10.69)	5 (3.82)	16 (12.03)	1 (0.75)
Back pain	14 (10.69)	0	14 (10.53)	1 (0.75)

*TEAE* treatment emergent adverse event, *SAE* serious adverse event, *TRAE* treatment-related adverse event, *ALT* alanine transaminase, *AST* aspartate aminotransferase, *ECG* electrocardiogram, *CK-MB* creatine kinase-MB, *GGT* serum gamma-glutamyl transferase, *LDH* lactate dehydrogenase, *PLT* platelet count, *WBC* white blood cell count

## Discussion

In the present randomized phase III trial, envonalkib significantly improved PFS compared with crizotinib (median, 24.87 vs. 11.60 months; HR = 0.47, 95% CI, 0.34–0.64). Moreover, envonalkib had superior efficacy over crizotinib against intracranial disease. In participants with brain target lesions at baseline, IRC-assessed CNS-ORRs were 78.95% and 23.81% in the envonalkib and crizotinib groups, respectively. Envonalkib significantly delayed disease progression in participants with baseline brain metastases and starkly reduced the risk of brain metastasis in participants without baseline brain metastasis. The incidence of TRAEs resulting in permanent discontinuation of envonalkib was low (6.11%), and no new safety signals were observed.

CNS metastases are protected from most systemic anticancer treatments due to the blood-brain barrier. Even small-molecule drugs can have a low diffusion rate through the barrier. As its lower capability of penetrating the brain-blood barrier, the activity against brain metastases of crizotinib is limited.^20^ Fortunately, the efficacy in controlling intracranial disease has been improved with second- and third-generation ALK inhibitors. Ceritinib, alectinib, brigatinib, ensartinib, and lorlatinib have produced intracranial response rates of 72.7%, 81%, 78%, 63.6%, and 82% in patients with measurable CNS lesions, respectively.^12–15,21^ In the present study, for participants with measurable intracranial lesions, IRC-assessed ORR was 78.95% in the envonalkib group, which was significantly elevated than that of the crizotinib group (23.81%) and similar to the IRC-assessed ORR of 70% observed in the phase I study.^19^ In addition, in participants without baseline brain metastasis, the IRC-assessed incidence rate of new brain metastases during the treatment was significantly reduced compared with that of the crizotinib group (2.15% vs. 11.70%, *p* = 0.0182). Collectively, these data strongly support the robust efficacy of envonalkib in controlling CNS disease. Controlling CNS metastases with systemic drugs is associated with lower morbidity than local treatments for CNS metastases (surgery and radiotherapy), and local treatments could then be kept as further options when the CNS disease progresses.

Previous phase III trials showed that second-generation ALK inhibitors, e.g., brigatinib, alectinib, and ensartinib, could significantly improve PFS in advanced ALK-positive NSCLC participants with no prior treatment with ALK inhibitors compared with crizotinib, with median PFS ranging from 25.8 to 34.8 months and HR values ranging from 0.43 to 0.51.^13,15,16,22,23^ In this study, using the same clinical setting as previous studies of second-generation ALK inhibitors, envonalkib also significantly prolonged PFS for ~13 months compared with crizotinib; IRC-assessed HR for disease progression or death was 0.47, which was comparable to those of other reported second-generation ALK inhibitors. In addition, although OS data for the present trial was immature, the 12- and 24-month OS rates in the envonalkib group were 90.6% and 78.4%, respectively, which were also similar to 84.3%–85% and 70.6%–78% reported in other second-generation ALK inhibitors.^12,14,15,21,22^

Older age and poorer performance status are prognostic factors in NSCLC.^24,25^ Moreover, the proportions of elderly patients (age >65 years) and individuals with poorer performance status (ECOG score of 1) were higher in the envonalkib group, but the clinical efficacy of envonalkib remained superior to that of crizotinib. Taken together, envonalkib could represent a new treatment option for patients with advanced ALK-positive NSCLC who had received no previous ALK-TKIs. Of note, lorlatinib also demonstrated outstanding clinical efficacy in this patient setting.^14^ Nevertheless, whether lorlatinib is an optimal option for first-line therapy is controversial since it plays an important role in salvage therapy for patients refractory to second-generation ALK inhibitors,^26^ as well as its uniquely unpleasant toxicity profile, such as hypertriglyceridemia, hypercholesterolemia, edema, peripheral neuropathy, and CNS toxicity.^27^

The safety profile of envonalkib from this phase III trial was generally consistent with that from the previous phase I study, with no new safety signal observed.^19^ The most common TRAEs included gastrointestinal symptoms (diarrhea, vomiting, nausea, and loss of appetite) and increased aminotransferase levels. These adverse events could be effectively managed by supportive care and dose reduction when necessary. Only 6.11% of the participants had to discontinue envonalkib treatment due to TRAEs, similar to the values reported for alectinib, brigatinib, and ensartinib (9.1–13%).^12,13,15^ In addition, no TRAEs leading to death were observed, indicating that envonalkib was generally well tolerated. Of course, safety data will have to be confirmed in future trials. Real-world studies are particularly useful in examining the occurrence of rare TRAEs.

The EML4-ALK fusion gene has variants that may have an impact on the treatment efficacy of ALK-TKIs.^28^ Indeed, different lengths of the EML4 gene can be fused with ALK. Variants 1 and 2 are unstable because of the exposure of the protein’s hydrophobic core, requiring a chaperone for avoiding misfolding, while variants 3a/b and 5 show higher stability. The stability of a given EML4-ALK protein variant is associated with the stability of the corresponding fusion protein, as well as inhibitor-induced protein degradation and drug sensitivity.^28^ Crizotinib treatment achieved a 2-year PFS rate of 76% for variants 1/2/others vs. 26% for variants 3a/b.^29^ On the other hand, such differences in efficacy against different EML4-ALK variants were not observed with alectinib in patients with NSCLC.^16^ Therefore, the impacts of the ALK variants on the efficacy of envonalkib will have to be examined. Indeed, TP53 mutations are risk factors for survival in NSCLC cases with ALK rearrangements administered crizotinib,^30^ and whether this is also true with envonalkib will have to be determined. The biomarker analysis of envonalkib will be reported in a separate manuscript in the future.

This study had limitations. Only Chinese participants were enrolled, indicating limited generalizability of the results. Because of the ethnic differences in the epidemiology and management of NSCLC,^31^ the clinical outcomes of envonalkib for advanced ALK-positive NSCLC in other ethnic cohorts need to be explored in future studies. Furthermore, although envonalkib appears to decrease the risk of CNS progression in participants with no baseline brain metastases, it will have to be verified in a properly powered clinical trial.

In conclusion, envonalkib is effective and with a manageably safe profile in advanced ALK-positive NSCLC patients who have previously received no ALK inhibitors, significantly improving PFS compared with crizotinib, as well as reducing the risk of progression or development of brain metastases. Envonalkib might be a new option for first-line treatment of advanced ALK-positive NSCLC.

## Materials and methods

### Study design and participants

This was a multicenter, randomized, open-label, active-controlled phase III trial, which enrolled patients with advanced ALK-positive NSCLC in 44 centers in China from August 21, 2019 to July 13, 2020. This trial was designed and conducted in accordance with the Declaration of Helsinki, GCP and current regulations. The study protocol was approved by the ethics committee of the leading center, and the study was registered with ClinicalTrials.gov (NCT04009317). All participants provided informed consent before study enrollment.

Inclusion criteria were: (1) age of 18–75 years; (2) ECOG PS score of 0–1; (3) life expectancy ≥12 weeks; (4) histologically or cytologically confirmed locally advanced or metastatic (stage IIIB–IV) NSCLC with ALK rearrangements; (5) no previous ALK inhibitors as systemic treatment for stage IIIB–IV NSCLC; (6) <2 lines of chemotherapy for stage IIIB–IV NSCLC; and (7) ≥1 measurable lesion(s) (other than brain lesions) in the screening period according to RECIST 1.1.

Key exclusion criteria were: (1) other malignant tumors; (2) allergy to the components of envonalkib or crizotinib; (3) other anti-tumor drugs administered within 4 weeks before the first dose; (4) any major surgeries performed within 4 weeks before the first dose; (5) received curative radiotherapy or minor surgery within 2 weeks before the first dose; (6) acute toxicity caused by any previous treatment not recovered to grade ≤1; (7) active infection; (8) uncontrolled congestive heart failure; (9) widely distributed interstitial fibrosis or interstitial lung disease; (10) symptomatic metastases to the central nervous system; or (11) any events preventing oral drug administration.

### Randomization and blinding

This was an open-label study. The participants were randomly assigned 1:1 to the envonalkib and crizotinib groups utilizing a central randomization system and additionally stratified according to brain metastasis status at baseline (yes vs. no) and the number of previous lines of chemotherapy (0 vs. 1).

### Intervention

The participants in the experimental group received envonalkib 600 mg BID, and the control group received crizotinib 250 mg BID in each 28-day cycle. In case of toxicity during the study, dose adjustment was considered to 500 mg BID and 400 mg BID in the envonalkib group and to 200 mg BID and 250 mg QD in the crizotinib group. In case that a third dose reduction was needed, the treatment was discontinued. Once the dose was reduced, it could not be increased again.

### Efficacy assessment

The primary endpoint was IRC-assessed PFS, according to RECIST 1.1, defined as the time from randomization to the first occurrence of disease progression or death from any cause, whichever occurred first.

The secondary endpoints included (1) investigator-assessed PFS, (2) IRC-assessed confirmed ORR, including the proportion of participants who achieved complete response (CR) or partial response (PR), (3) disease control rate (DCR), including the proportion of participants who achieved CR, PR and stable disease (SD), (4) OS, defined as the time from randomization to death from any cause, (5) IRC-assessed DOR, defined as the time from the first date of documented CR or PR to the first onset of disease progression or death from any cause, whichever occurred first, (6) IRC-assessed confirmed CNS-ORR, which was defined as the proportion of participants achieving intracranial CR or intracranial PR among participants with baseline intracranial target lesions assessed by RANO-BM criteria, (7) CNS-DOR, which was defined as the duration of CNS remission in participants with baseline intracranial target lesions, (8) CNS-TTP, which was defined as the time from randomization to the first onset of CNS disease progression (not including death) in participants with baseline brain metastases and participants with baseline intracranial target lesions, and (9) health-related quality-of-life scores, including EORTC QLQ-C30, EORTC QLQ-LC13, EuroQol EQ-5D and visual analogue scale (VAS) scores.

The exploratory endpoints included tumor mutational status at baseline and after disease progression, mutations of ALK and other genes, and the rearrangement status of circulating tumor nucleic acids in plasma.

### Safety evaluation

TEAEs were recorded and graded using the CTCAE 5.0 criteria. SAEs were defined as TEAEs resulting in death, hospitalization or prolongation of hospitalization, life-threatening consequences, permanent or severe disability/loss of function, or congenital malformations/birth defects. Non-serious adverse events of special interest included potential drug-induced liver injury and interstitial lung disease or pneumonitis.

### Statistical analysis

The sample size was calculated with PASS 16.0. The estimated median PFS of participants with treatment-naive advanced ALK-positive NSCLC administered crizotinib monotherapy was 11 months; the estimated PFS was 5.67 months longer in participants administered envonalkib compared with crizotinib, indicating an HR of 0.66. The randomization ratio of the two groups was 1:1; *α* was 0.05, and the statistical power was 80%. The log-rank test was utilized to compare the PFS between the two groups. The estimated enrollment time was 18 months; the follow-up time was 18 months, with an estimated dropout rate of 15%. Totally 183 PFS events were required; thus, a sample size of 260 participants (130 per group) was required. Interim analysis was conducted upon observation of 70% of PFS events (128 events). The O’Brien-Fleming approach in the Lan-DeMets α-spending function was used to control the overall Type I error, and the nominal significance level of the first interim analysis was 0.01477.

The ITT population included all randomized participants and was used in the efficacy analysis. The SS included all participants administered with the study drug at least once and whose safety assessments were available and was used for safety analysis.

Continuous data were presented as mean ± standard deviation or median and interquartile range (IQR) and compared between the two groups by independent samples *t*-test or nonparametric test. Categorical variables were presented as number (percentage) and compared between groups by the chi-square test or Fisher’s exact test. PFS and DOR were compared between groups by the stratified log-rank test. HRs and their 95% CIs were obtained by Cox proportional hazards regression models, and adjusted HRs were calculated according to the stratification factors. The results of the unstratified log-rank test and Cox proportional hazards regression models were also presented. Survival was assessed using Kaplan–Meier curves; ORRs were compared by the Cochran–Mantel–Haenszel method, and the Fisher’s exact test was performed to calculate ORRs and their 95% CIs. SAS 9.4 was utilized for data analysis, with two-sided *p* < 0.05 indicating statistical significance.

### Supplementary information

Supplementary materials

## Data Availability

The authors confirm that the data supporting the findings of this study are available within the article and its Supplementary Materials.
