# Supplementary material for: Envonalkib versus crizotinib for treatment-naive ALK-positive non-small cell lung cancer: a randomized, multicenter, open-label, phase III trial
Source: Signal Transduct Target Ther. 2023 Aug 14;8:301. doi: 10.1038/s41392-023-01538-w (PMC10423717; doi:10.1038/s41392-023-01538-w)
Supplement: Supplementary file 1 — Supplementary materials [file 41392_2023_1538_MOESM1_ESM.docx]

# Supplementary Materials for

# Envonalkib versus crizotinib for treatment-naive ALK-positive non-small-cell lung cancer: a randomized, multicenter, open-label, phase III trial

Yunpeng Yang, Jie Min, Nong Yang, Qitao Yu, Ying Cheng, Yanqiu Zhao, Manxiang Li, Hong Chen, Shou’an Ren, Jianying Zhou, Wu Zhuang, Xintian Qin, Lejie Cao, Yan Yu, Jian Zhang, Jianxing He, Jifeng Feng, Hao Yu, Li Zhang, Wenfeng Fang

Correspondence to: zhangli@sysucc.org.cn; fangwf@sysucc.org.cn

**This PDF file includes:**

Figures. S1 to S2

Tables S1


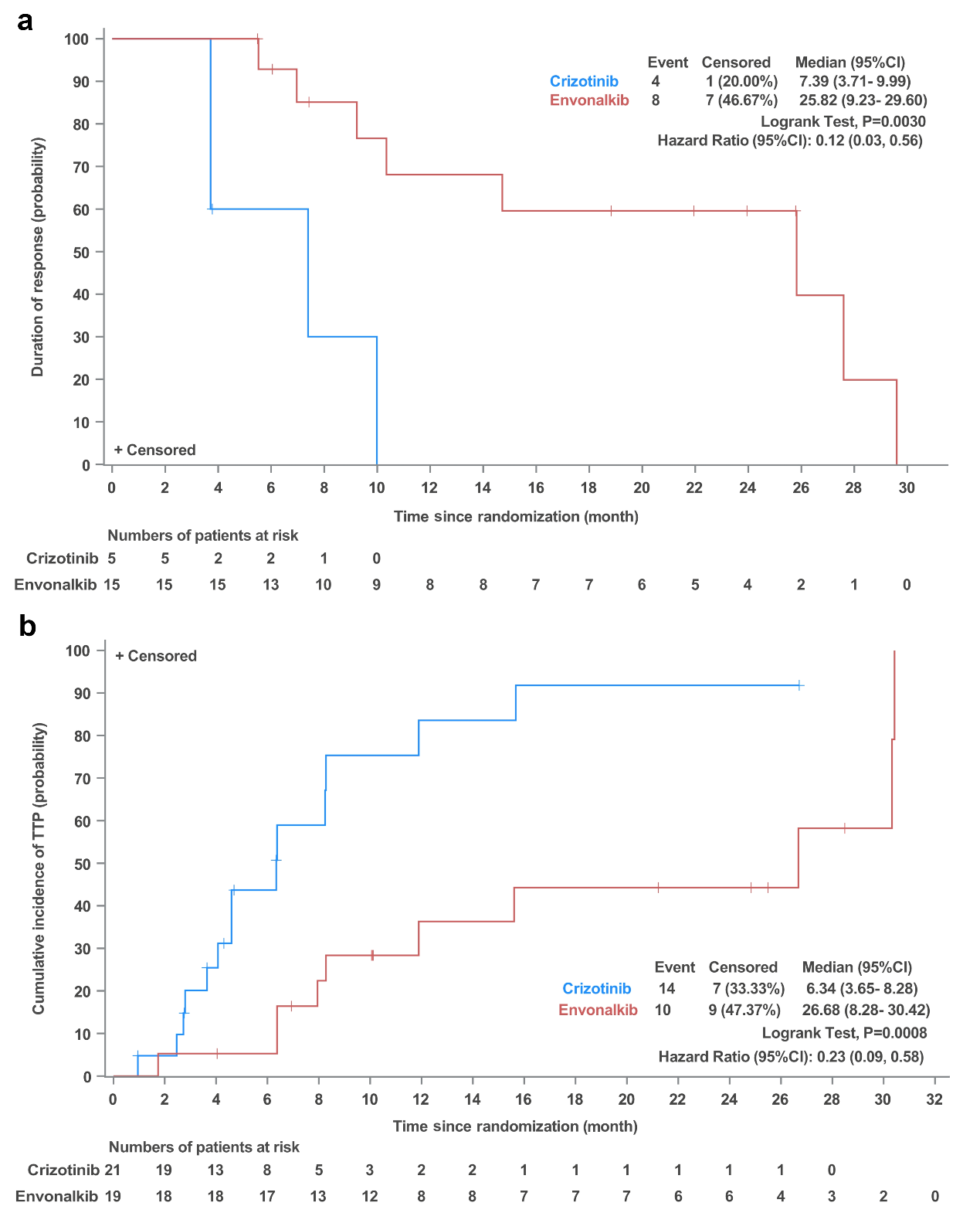


**Figure. S1.**

Independent review committee-assessed duration of response (a) and cumulative incidence of central nervous system (CNS) progression (b) in patients with baseline intracranial target lesions.


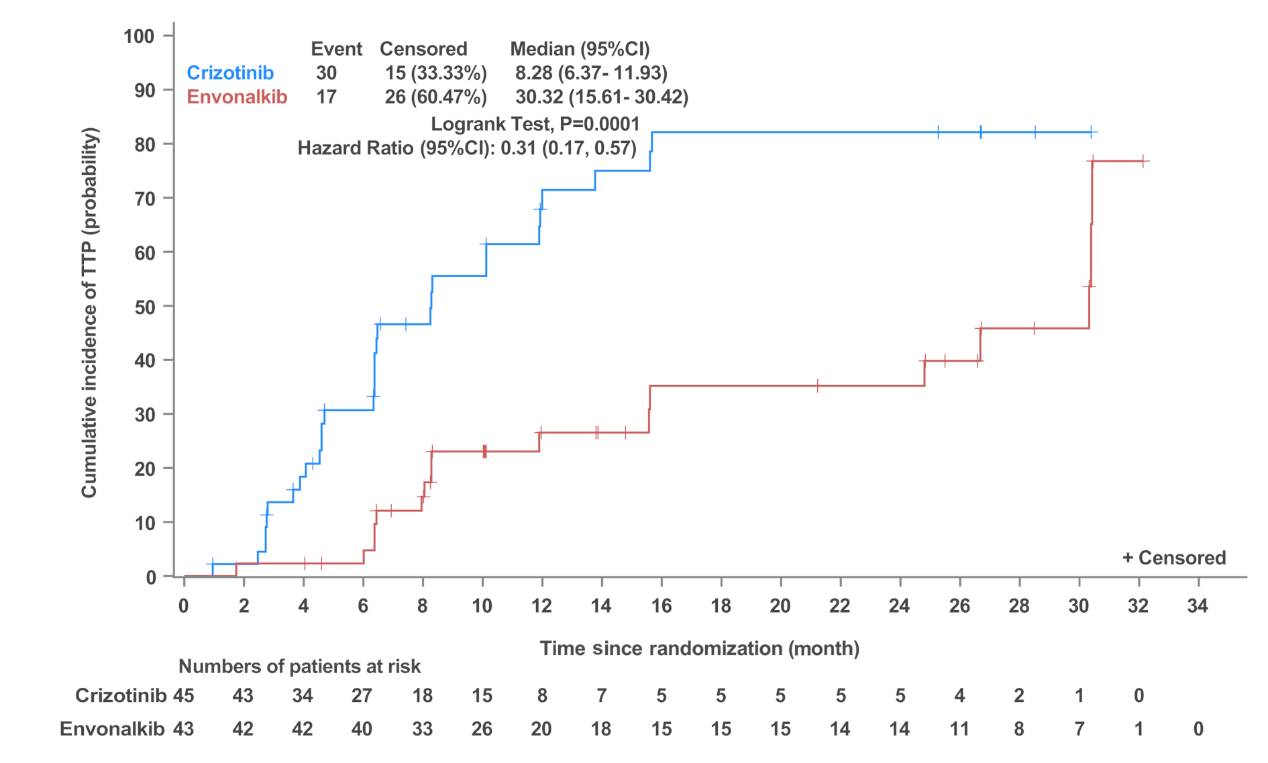


**Figure. S2.**

Independent review committee-assessed cumulative incidence of central nervous system (CNS) progression in patients with baseline intracranial lesions.

**Table S1.**

Objective response assessed by independent review committee among patients with intracranial target lesions at baseline

|  | Envonalkib (N=19) | Crizotinib (N=21) |
| --- | --- | --- |
| CR | 0 | 1 (4.76) |
| PR | 15 (78.95) | 4 (19.05) |
| SD | 3 (15.79) | 11 (52.38) |
| PD | 1 (5.26) | 4 (19.05) |
| NE | 0 | 1 (4.76) |
| **Confirmed ORR** | **15 (78.95)** | **5 (23.81)** |
| 95% CI | 54.43−93.95 | 8.22−47.17 |
| P value | 0.0012 | |

ORR: objective response rate; CR: complete response; PR: partial response; SD: stable disease; PD: progressive disease; NE: could not be evaluated; CI: confidence interval.
